# The Cyclopædia of Anatomy and Physiology.—Articles, Nerve, Nervous System, and Nervous Centres

**Published:** 1845-10

**Authors:** 


I. THE CYCLOPÆDIA OF ANATOMY AND PHYSIOLOGY, Parts 25 and 26. 1844. Articles:—Nerve, Dr. *Todd;* Nervous System, (Comparative Anatomy), by *J. Anderson,* Esq. Nervous Centres, Dr. *Todd.*

II. THE PHYSIOLOGICAL ANATOMY AND PHYSIOLOGY OF MAN. By *R. B. Todd,* M.D., F.R.S., and *William Bowman,* F.R.S. Part II. 1845.

III. TRAITÉ COMPLET DE L'ANATOMIE, DE LA PHYSIOLOGIE, ET DE LA PATHOLOGIE DU SYSTEME NERVEUX CÉRÉBRO-SPINAL. Par *M. Foville.* 1re Partie. Anatomie. Paris 1844.

IV. ON THE NERVOUS AND CIRCULATING SYSTEMS IN MYRIAPODA AND MACROUROUS ARACHNIDA. By *G. Newport,* F.R.S. Philosophical Transactions, 1843.

V. PHYSIOLOGISCHE UNTERSUCHUNGEN ÜBER DIE BEWEGUNGEN DES GEHIRNS UND RÜCKENMARKS, INSBESONDERE DEN EINFLUSS DES CEREBROSPINALFLÜSSIGKEIT AUF DIE SELBEN. Von *A. Ecker.* Stuttgart, 1843.

VI. UEBER DEN VERLAUF DER NERVENFASERN IM RÜCKENMARKE DES FROSCHES VON Dr. *Julius Budge.* ERFAHRUNGEN ÜBER DIE FUNCTIONELLE SELBSTANDIGKEIT DES SYMPATHISCHEN NERVEN SYSTEMS, von *F. Bidder.* VORLAUFIGE MITTHEILUNG ÜBER DIE STRUCTUR DER GANGLIEN UND DEN URSPRUNG DER NERVEN BEI WIRBELLOSEN THIEREN. Von Dr. *Friedrich Will.* Muller's Archiv. 1844.

VII. LEHRBUCH DER PHYSIOLOGIE DES MENSCHEN. BAND. II. Von Dr. *G. Valentin.* 1844.

VIII. RECHERCHES EXPÉRIMENTALES SUR LES FONCTIONS DU NERF SPINAL. Par *C. Bernard.* Archives Gén. de Medicine. 1844.

IN resuming our notice of the Nervous System, we shall, in the first place, allude to the membranes which invest the cerebral and spinal centres. To the medical practitioner the most interesting of the meninges is the arachnoid; and of this membrane, its relation to the pia mater and the cerebrospinal liquid is the most worthy of notice. This fluid, first noticed by Cotunnius, has in late years been admirably investigated by Magendie, from whose work, extended extracts, accompanied by most characteristic wood-cuts, are given by Dr. Todd in the Cyclopædia of Anatomy. It is important to know that the seat of the secretion is not within the sac of the arachnoid, but in the very lax tissue uniting that tunic to the pia mater, hence called *sub-arachnoid.* It is apparently derived from the vessels of the pia mater, and is so very abundant, that even in health as much as five ounces was drawn off by Cotunnius, by means of a puncture made in the lumbar region. This estimate is greater than that of Magendie, who regards the minimum quantity in the adult as two ounces, though in old age the proportion is so much increased, that, in the aged poor, he has found eight, ten, and even twelve ounces.

The exact relations of the sub-arachnoid space are of considerable interest, both in a physiological and pathological point of view. And first of all it may be well to point out that this space is uninterruptedly continuous from the upper surface of the cerebrum along the whole extent of the cord to the extremity of the sacrum, or, in other words, that the cranium and the vertebral canal form in life, as it is familiarly known they do in the skeleton,—one continuous cavity. The most difficult question to be resolved is, whether there be an opening at the calamus scriptorius, leading from the interior of the 4th, and thus indirectly from the lateral ventricles, into the sub-arachnoidean space. Such a communication, by which the cerebro-spinal fluid continually passes, either to enter the ventricles or to issue from them, is admitted by Magendie under the denomination of "*orifice des cavités encephaliques;*" a similar view is taken by Ecker, whilst Dr. Todd conceives that this orifice does not exist naturally, but that the 4th ventricle is closed in the same way as the inferior horn of the lateral ventricles, the aperture described by Magendie being the result of rupture, either caused by the violence to which the brain is subject in its removal, or in the subsequent manipulation. We incline ourselves to this last view, and among other reasons, for this, that no such instance of the perforation of a membrane is known in any other part of the body.

The general use of this secretion is defensive; indeed, it is impossible to conceive of any mechanism more perfect than that of the sub-arachnoid liquid and its associated structures. The brain, and more especially the cord, are, like the fœtus in utero, suspended in a fluid, which, receiving the various concussions to which the cranium and the spine are subject, diffuses the shock throughout its whole extent, and, by thus dividing, weakens, or entirely obviates, the injurious effects. But, besides these external sources of disturbance, the delicate nervous centres are momentarily exposed to variations in the movements of the surrounding vascular currents, dependent upon the alternating actions of the heart and respiratory muscles. Thus, it is well known that, when the brain is observed in the infant through the fontanelle, or in the adult through the aperture made by the trephine, two distinct motions are seen, each of which, when carefully examined, is double: one class corresponds to the actions of the heart, the brain being raised at the time of the systole and depressed at the instant of the diastole; the second class accompanies the respiratory motions, the cerebrum rising with expiration and sinking with inspiration. In these phenomena, and particularly in those of the respiratory order, according to Dr. Ecker, who has made many experiments upon various animals to elucidate the subject, the cerebro-spinal liquid performs an important part. He found, upon laying bare the occipito-atloidean ligament, and making pressure upon it, so as to force the fluid from the vertebral to the cranial cavity, that the brain rose upwards; whilst, upon removing the larger portion of the fluid, by puncturing the ligament, the brain sank. After this latter operation, the respiratory motions, even when forcibly excited, produced little or no corresponding movements of the brain; but when the wound was closed, and time allowed for the reproduction of the sub-arachnoid fluid, the elevation and sinking of the brain again returned. The explanation of Dr. Ecker is, that, during expiration, the blood, driven into the arteries in the centrifugal direction, and prevented returning in its centripetal course, accumulates in the sinuses as well as in the capillaries of the nervous centres; that it thus displaces the cerebro-spinal fluid towards the base of the brain; and that this, to escape the pressure, enters into the fourth ventricle by the foramen of Magendie, streams into the lateral ventricles by the aquaduct of Sylvius, and so raises up the cerebral hemispheres. The elevation of the brain, which corresponds to the systole of the left ventricle, is attributed to the cause assigned by physiologists in general, namely, to the reception of a larger quantity of blood by the carotid and vertebral arteries and their branches. Some interesting peculiarities were observed in connexion with the spinal cord; thus, the motions dependent on the heart's actions were much weaker than in the case of the brain, and those resulting from the respiratory movements were not only more simple in character, but persisted after the cerebro-spinal fluid was drawn off. This latter circumstance is attributed to the powerful distension in expiration of the rich venous plexuses and sinuses of the vertebral canal.

Our readers will perceive that these valuable researches of Ecker have an immediate bearing upon the views of Dr. Abercrombie, respecting the circulation within the cranium. They corroborate the conclusions arrived at by Dr. G. Burrows upon other grounds, that the quantity of blood does vary, and that in a most important manner, in the cranial cavity. It is our opinion, however, that Ecker is in error when he assumes that the cerebro-spinal fluid recedes into the ventricles during the expiratory action; we rather believe that at this period a portion of the fluid, corresponding in quantity exactly to the additional amount of blood accumulated within the skull, passes into the vertebral canal, where the pressure, partly taken off by the yielding of the numerous tubular sheaths around the nerves, which we regard as the safety-valve of the whole cerebro-spinal apparatus, and partly broken in its effects by the extent of the canal, causes no injurious results.

We cannot better conclude our notice of this important mechanism, which, in consideration of its immediate bearing upon the pathology of congestion, of effusion, and even of extravasation, has been more extended than we originally designed, than by quoting the following practical observations of Dr. Todd and Mr. Bowman.

"This fluid is a valuable regulator of vascular fulness within the cranium, and a protector of the brain against too much pressure from within. So long as it exists in normal quantity, it resists the entrance of more than a certain proportion of blood into the vessels. Under the influence of an unusual force of the heart, an undue quantity of blood may be forced into the brain; the effects of which will be, first, the displacement of a part, or of the whole surrounding fluid, and, secondly, the compression of the brain.

"On the other hand, the brain may receive too little blood. In such a case, if the surrounding fluid do not increase too rapidly, the requisite degree of pressure will be maintained, and the healthy action of the brain preserved. But, if the brain be deprived of its due proportion of blood by some sudden depression of the heart's power, there is no time nor source for the pouring out of new fluid, and a state of syncope, or of delirium, will ensue. Such seems to be the explanation of those cases of delirium which ensue upon hæmorrhages, large bleedings, or the sudden supervention of inflammation of the pericardium or endocardium."—*Physiological Anatomy,* p. 298.

Of the Spinal Cord.—With the exception of the volume of M. Foville, which is the first part of a complete treatise on the anatomy, physiology, and pathology of the cerebro-spinal system, we do not find, in the works before us, any very novel facts relating to this nervous centre, so far at least as human anatomy is concerned. The description by M. Foville, in addition to a full account of what was already known, also contains several new facts. Thus, besides the antero-lateral and posterior columns admitted by all anatomists, M. Foville has represented, in the excellent plates accompanying his work, two narrow fasciculi which, continuous superiorly with the posterior pyramids of the medulla oblongata, run downwards close on the outer side of the posterior median fissure, and thus "extend from the calamus to the lumbar extremity of the cord."—(*Traité Complet. de l'Anat. &c., p.* 283.) Several writers, Haller, Rolando, Chaussier, and Mayo, had previously noticed a small fasciculus proceeding from the posterior pyramids, but then it was usually asserted that it was confined to the cervical part of the cord, or, according to Mr. Mayo, to the superior two-thirds: Cruveilhier, however, distinctly affirms that these small cords are prolonged throughout the whole length of the medulla spinalis, and with this concurrent testimony the existence of such a structure can be no longer doubtful. Another, and till this time unknown, fasciculus, is a narrow band of fibrous matter, placed on the lateral aspect of the cord, along the whole of which it extends; superiorly it passes between the corpus olivare and c. restiforme, and then, which is an interesting fact, joins the crus cerebelli, and thus ultimately penetrates into the cerebellum.—(*L. c., p.* 285. *and Planche* 1, *fig.* 3.)

The connexions of the various parts of the cord—a point of considerable importance in reference to the actions of this organ—are carefully investigated by M. Foville. The most important results are as follows: all the white masses, which enter into the formation of the cord, resolve themselves into longitudinal fibres, which are disposed nearly in the same manner in all the columns or fasciculi; the anterior column is intimately united by fibres, similar to those of the column itself, to the anterior commissure, which thus serves to connect together the two anterior columns; the posterior column seems to be united by a fusion of substance with the central gray axis of the cord, and is also united with the posterior white commissure; it is not certain whether any white fibres pass from the lateral column to the internal axis of the cord; the anterior white commissure is not simply an apparatus of connexion, but is composed of fibres which, derived as stated above from the anterior columns, really decussate; lastly, the gray substance is "formed of fibres interlaced with an enormous number of blood-vessels, and, as it were, sprinkled with a reddish-gray powder, to which it owes its colour."

These details indicate, what is seen in all the other nervous centres, a most intimate union, namely, between the fibrous and the vesicular or gray substance, a disposition which is doubtless essential to innervation. The account formerly given by Rachetti and Rolando of this arrangement is so clear, that we are induced to transcribe the following passage, taken from Weber's Anatomy: "Rachetti remarks that the white substance is divided into layers by gray lines, which run outwards from the gray centre. The same disposition is noticed by Rolando, who states that the fibrous substance consists of a folded medullary membrane, the convoluted edges of which are turned alternately towards the centre and towards the periphery, and run through the whole length of the cord: between the layers of these folds, thin processes of the pia mater penetrate from without, and delicate laminæ of the gray substance from within." Foville notices a disposition of the white fibres presenting a radiate appearance in the transverse section, evidently depending on the vasculo-cellular septa above-described, converging from the circular periphery of the cord towards the centre.

A question which, though of little consequence, has commanded much attention, relates to the existence of a central canal in the spinal cord. Stilling and Wallach admit this formation and place it in the gray substance, whilst Dr. Todd regards it rather as being the patulous mouth of a blood-vessel. Foville says there is a central canal, or ventricle as he terms it, constantly existing in vertebrate animals, and also in man at an early age; he has also met with it accidentally at all other epochs of life, but in such cases its demonstration is difficult. We are convinced that there is in young animals a distinct central passage, which is readily displayed by Stilling's method, that of making very thin transverse sections: but we are doubtful as to its persistence in the fully-formed cord.

ORIGIN OF THE SPINAL NERVES.—This has become, since the great discovery of Bell, a point of primary importance; and yet, although it would seem as if we were approaching a solution, and although some of the works named in the present article have undoubtedly given us some new facts, the exact relations of the roots of the spinal nerves with the cord, are not satisfactorily made out.

Dr. Todd thus alludes to the opinion of Mr. Grainger, that each root of the spinal nerves divides into two sets of fibres, one penetrating into the gray substance, and the other becoming continuous with the white fibrous structure.

"I have repeated the dissections of the two roots of the nerves in the manner described by Mr. Grainger, and am enabled to confirm his general results. It appeared to me, however, that considerably the greater number of the fibres passed in at right angles, whilst those which might be supposed to take an upward course were few and indistinct, and seemed rather to pass obliquely inwards and slightly upwards than to approach the vertical direction. In short, when the fibres had penetrated the medullary substance, they seemed to diverge from one another,—those which occupied a central position preserving much more of parallelism than either the upper or the lower ones. It is extremely difficult to demonstrate the direct continuity between the fibres of the nervous roots and those of the cord."—*Cyclop. of Anat.*, p. 660.

In the somewhat more recent work of this gentleman and Mr. Bowman (*Physiological Anatomy*), an opinion, in some respects, opposed to the above is advanced. In alluding to the several hypotheses which have been advanced respecting the nervous agency ministering to the sensori-volitional and excito-motory phenomena, these writers thus express themselves.

"The third hypothesis appears to us to admit of fewer objections than either of the others, and to be more consonant with what seems to be the correct anatomy of the cord. It supposes that the mechanism of a mental and that of a physical nervous action are essentially the same, differing only in the nature and the mode of application of the stimulus. The same *afferent* and *efferent* fibres are exerted in the one case as in the other; the former acting as *sensitive* or *excitor*, or both; the latter as channels for *voluntary, emotional,* or strictly *physical* impulses to motion.

"This hypothesis is content to assume that fibres of sensation and voluntary motion do not pass beyond that particular segment of the cord with which they are connected; and that each segment of the cord communicates readily with the brain through the horns of gray matter, or through commissural fibres which pass between the segments of the cord, and from the upper segment of the latter to the brain. The anatomy of the cord, so far as our present knowledge extends, is favourable to this hypothesis, for it is much more probable that all the roots of the spinal nerves are implanted in their proper segments of the cord, than that some pass up to the brain, and others remain in the cord." P. 329.

The account given by M. Foville of the origin of the spinal nerves is not very minute; he affirms, however, as clearly demonstrated by observation, that "the nerves of the posterior fasciculus (these are the posterior roots of other anatomists) plunge by some of their roots into the gray substance of the posterior fasciculus, and unite by other roots with the fibrous substance of this same fasciculus."—(*L. c., p.* 500.) Further on it is stated, the anterior roots are similarly connected in part with the fibrous, in part with the gray substance. This description of the nerves in man corresponds with the researches of Budge into those of the frog, noticed in our last number (Med. Chir. Rev., p. 14), so far as relates to the connexion with the fibrous and gray substances. Professor Valentin contends for the perfect union of the nerve-fibres with those of the cord, a junction which he has depicted in his earlier work on the nerves, and which he thus describes in his Physiology:—

"Microscopical investigation teaches that the primitive fibres of the sentient and motor roots are directly continued into the corresponding fibres of the spinal cord; that they in this place decrease in size; and that they then ascend in different ways towards the brain;" and a little further on he lays it down as an essential point, that "the spinal cord takes up or receives into itself successively all the sentient and motor roots of the individual nerves, and transmits them to the medulla oblongata."—(*L. c., p.* 738.)

The conclusion we draw from all these investigations and from our own examination is, that each root of the compound spinal nerve is continuous by some of its fibres with the white fibres of the cord, and by others, with the gray substance; and we may further add that, in this view, we are corroborated by the opinion of several excellent observers.

Of the Encephalon.—It would not be doing justice to Dr. Todd, if we did not call attention to the excellent description he has given of the structure of the brain; indeed, we have no hesitation in saying, that the several articles relating to the nervous system contributed by this gentleman in the Cyclopædia of Anatomy, and which bear the impress of great labour and research, embody all that is known upon this comprehensive subject, and may be safely relied upon by those who are debarred from referring themselves to the original authorities. Some interesting tables are given to show the relative and absolute weight of the encephalon, and which, being based upon extended data, especially those of Dr. J. Reid, are probably more approximative to the truth than former calculations. From these calculations it appears that the average weight of the encephalon, between the ages of 25 and 55 years, is in the male 50 oz. $3\frac{1}{2}$ dr. (avoirdupois),—in the female 44 oz. $8\frac{1}{2}$ dr., giving a difference in favour of the male of 5 oz. 11 dr.; the average weight of the cerebrum is in the male 43 oz. $15\frac{3}{4}$ dr.—in the female, 38 oz. 12 dr.; of the cerebellum, male 5 oz. 4 dr.,—female 4 oz. $12\frac{1}{2}$ dr.; of the pons and medulla oblongata about one ounce. Tiedemann, in reference to this difference in the two sexes, observes that—

> "Although Aristotle has remarked that the female brain is absolutely smaller than the male, it is nevertheless not relatively smaller compared with the body; for the female body is, in general, lighter than that of the male. The female brain is for the most part even larger than the male, compared with the size of the body."—*Cyclop. of Anatomy*, p. 665.

The large comparative size of the brain in infants and young children is well known; and it is interesting to observe that, before the sexual desires are developed, the cerebrum appears to be absolutely larger in the male than in the female sex: thus, from one to four years, the encephalon in the male weighs 39 oz. $4\frac{2}{5}$, dr. in the female 37 oz. 9 dr. A decided diminution in the average weight of the brain was noticed by Dr. Reid in females above 60 years of age; but among males, this was not apparent until a later period. This change is accompanied with an increase in the quantity of the cerebro-spinal fluid, which particularly accumulates in the sulci between the atrophied convolutions, as it also does in the brains of people in the prime of life, who have for some time been addicted to excessive indulgence in ardent spirits. Dr. Todd regards this accumulation, and we think with justice, as being of a conservative kind; and adduces it as a warning to practitioners, not to attribute too much importance to liquid effusion upon the brain and spinal cord, as a cause of morbid pressure.

It appears that the brain of Cuvier weighed as much as 59 oz. 4 dr., and that of Dupuytren 59 ounces troy weight; but it is well to bear in mind, that both these eminent men died with the brain in a diseased state. The general result of Tiedemann's well-known observations on the relative capacity of the skull (ascertained by first weighing the cranium and then finding out the quantity of millet seed contained within it) is, that the brain of the Negro is upon the whole quite as large as that of the European and other races; the examples of skulls of the smallest capacity were found among Hindoos and Americans. Other evidence leads to the same conclusion, and it may be well to notice that the measure of the facial angle, as suggested by Camper, which appeared to give opposite results, has been entirely abandoned by physiologists as a test for measuring the actual development of the brain.—(*L. c.* 662 *et seq.*)

*Cerebral Convolutions.*—In the Physiological Anatomy there is given an interesting sketch, principally taken from the observations of M. Leuret, relating to the arrangement of the convolutions. It is certain, notwithstanding the apparent confusion, that these important organs, for as such they ought to be regarded, observe a definite order, their disposition being determined by certain leading or typical convolutions which have been long known to anatomists, although there is some discrepancy in the accounts given of their number and disposition. Foville arranges the convolutions in the human brain in four classes. 1. A single convolution lying upon the corpus callosum and winding around the borders of it to the base of the cerebrum; it is called by Foville *l'ourlet* (hem or border). 2. This class comprises two convolutions, which proceed from the locus perforatus run forwards, then upwards and backwards, one along the inner border of the hemisphere, the other at some distance on the outer side of the last, and so reach the posterior edge of locus perforatus. Thus, it is seen that "the single convolution of the first order and the two of the second class, are all great lines irregularly circular, situated in an antero-posterior and nearly vertical plane, and united to the limits of the locus perforatus." 3. These serve as a means of union between the convolution of the first order and the two of the second. 4. These, irregularly disposed on the convexity of the hemisphere, fill up the interval of the two convolutions of the second order.—(*L. c.* p. 191).

According to Leuret, the term of convolution ought to be confined to the primary formations, the smaller ones forming angles with them, being in his estimation, mere folds derived from the leading convolutions and connecting them together. It was affirmed by Gall and Spurzheim—" that the presence and number of the convolutions are in direct relation to the volume of the brain, but Leuret has shown that such is far from being universally the case." An extended inquiry into comparative anatomy confirms this assertion, and distinctly proves that the principle regulating the development of the convolutions relates not to the absolute or even relative size of the cerebrum, but to the rank of the animal in the zoological scale, which last may perhaps be assumed as measuring in a general way the grade of intelligence. Thus, whilst in animals below the mammalia the hemispheres are perfectly smooth, it is found that the convolutions begin to appear, though in a simple form, in the monotremata and marsupiata, and increase in number and complexity in ascending to the ruminants, carnivora and quadrumana.

The minute structure of the convolutions displays a more complex character than is generally known. These bodies, for example, are not exclusively formed, as it is often supposed, of the gray substance only: there enters into the composition of all, but in varying degrees, the fibrous matter, which in some regions presents an intricate disposition. The authors of the Physiological Anatomy give the following description:—

"That portion of the gray layer which is in contact with the pia mater is purely vesicular, *i. e.* unmixed with nerve-tubes, with the exception of a few stray ones on the surface; but blood-vessels penetrate it in very great numbers. The more deeply-seated portion, however, contains very numerous tubular fibres, which become larger as they approach the white matter. It is very plain, that a large proportion of the constituent fibres of the white matter of the convolutions penetrate the gray matter: these appear to enter it more or less at right angles to that portion of the gray surface with which they are more immediately in relation; and, on the other hand, they converge inwards towards the central parts of the brain, the corpora striata and optic thalami. A large proportion, therefore, of the white substance of the hemispheres, the *centrum ovale*, consists of fibres which establish a communication between the gray undulating surface and these central gangliform bodies."—*L. c.* p. 283.

Remak, who is well known as a minute investigator of the nervous system, has also briefly described and represented the texture of the convolutions. According to this writer, there are, in each of these organs, not less than six different layers, consisting of three very thin laminæ of white substance, following the curves of the convolutions, one of which is placed on the exterior, forming a kind of cortex; and of three interposed layers of gray matter, abounding in ganglionic globules or vesicles. In addition to these plates there are the fibres radiating from the interior of the hemispheres, and which, according to Remak, lose themselves by degrees in the most external of the gray layers. (*Müller's Archiv*, 1844, p. 468, and Taf. XII). When to all this elaborate structure we add, that an immense quantity of blood incessantly streams through the convolutions, we shall be enabled to form some conception of the kind of instrument in which the nervous force is generated, or at all events through which it is displayed.

The only work which contains any new researches into the anatomy of the brain is that of M. Foville, and indeed many of the opinions therein advanced have already appeared in various publications. The views of M. Foville, which are in many essential points different from those of other authorities, have commanded considerable attention among his compatriots, but we are not aware that any English writer has adopted them; they are, however, so important, and, if confirmed, would so entirely modify received opinions, that we propose, now that they are brought forward in a finished form, to lay them before our readers as much in detail as our limits will permit.

M. Foville's first researches were communicated to the Academy of Medicine in 1825; but we shall avail ourselves of his article "*Encéphale*" (contained in the Dictionnaire de Med. et de Chir. Pratiques, 1831), to indicate the first steps of the inquiry. The author there affirms that each peduncle of the brain (crus cerebri) is composed of two distinct layers, one of which, the anterior inferior, is the continuation of the anterior pyramid of the medulla oblongata, which it is well known traverses the pons Varolii; the other, or posterior fasciculus, is in like manner the continuation of a part of the medulla oblongata posterior to the anterior pyramid, which passes above the fibres of the pons and forms the floor of the 4th ventricle. These two layers, closely approaching each other, but still kept distinct by the interposition of the locus niger, penetrate, the anterior into the corpus striatum, the posterior into the optic thalamus, and radiating in these bodies, reach their outer border: here a most important disposition commences, by their dividing into three planes perfectly distinct from each other, an internal, a superior, and an inferior. The internal plane, called the ventricular, turns upwards and then curving inwards towards the median line, forms, by its union with that of the opposite side, the corpus callosum. The superior plane, called that of the hemisphere, passes at first upwards parallel with, and back to back (*adossé*) to that of the corpus callosum, but at the point where the latter bends inwards, this plane passes nearly vertically to reach the gray substance of the convolutions, in the whole length of the most elevated part of the hemisphere. The inferior plane descends around the exterior part of the corpus striatum, surrounds this body inferiorly, and, approaching the median line, mounts upwards in juxta-position with the corresponding plane of the other side to form by their union the septum lucidum.

In the work lately published, and of which the title is inserted at the head of this article, M. Foville has gone into a much more detailed and very interesting account of the fibrous and other parts of the brain. The fibres of the anterior pyramid, which we have seen pass to the hemisphere, are stated there to divide into two planes, both destined exclusively to the convolutions which form the superior and convex part of the hemisphere, and which belong especially to the second class already enumerated. The fibres emanating from the posterior part of the medulla oblongata equally with the last divide into two planes, of which the superior and larger advances into the corpus striatum, and then curves upwards to form the corpus callosum. The inferior plane, although the smaller, has extensive and important ramifications: passing from the centre of the thalamus, it winds in such a manner as to surround with a complete and very remarkable ring the ascending fasciculus from the anterior pyramid; turns in front of the lateral part of the foramen of Bichat, and at length, having furnished roots to the optic and olfactory nerves, forms between the anterior and middle lobes a white space called perforated quadrangle (*quadrilatère perforé*). This space forms a kind of centre, from which proceed numerous arciform fibres, constituting a certain number of fibrous circles, surrounding the plane of the crus cerebri derived from the anterior pyramid, and terminating in a peculiar manner in the hemisphere at the same perforated space. Foville thus enumerates these circles or rings:—1. Tænia Semicircularis. 2. An analogous band to the last, not before known, which surrounds the outer border of the corpus striatum as the other does the thalamus. 3. The half of the formix and corpus fimbriatum. 4. The median longitudinal band of the corpus callosum. 5. The remarkable fibrous band called l'ourlet. Prior to the publication of Foville's late researches, M. Gerdy had recognised and described the annular disposition of those parts of the brain which crown the superior portion of the crus cerebri. He admitted the parts just enumerated, but also included the corpus callosum and even, which is clearly erroneous, the tela choroides.

From his investigations Foville draws several important anatomical deductions. With respect to the convolutions, however numerous, they form but two groups: one set occupying, as we have seen, the convex surface of the hemisphere, crown the top of the ascending fibres of the anterior pyramids, and are therefore in relation with the anterior part of the spinal cord, and with the anterior roots of the nerves; the other set, consisting especially of the "ourlet," the convolutions on the flat surface next the falx major, and those forming the *insula*, are placed on the course of the fibres coming from the posterior part of the medulla oblongata, and are continuous not only with the posterior roots of the spinal nerves, but with the sentient cerebral nerves, the olfactory, the optic, and the acoustic. He further holds as a fundamental position that " all parts which enter into the composition of the cerebrum either proceed from, or return to, the peduncular region;" and moreover, that " whatever parts be examined, they present two directions, two forms altogether distinct, of which the different groups of convolutions offer the types." This disposition is also evidenced in the fibres, of which one set are *circular*, seen in the fibrous ribbon of the "ourlet;" and the other set *radiating*, seen in those expanding in the hemisphere. The author remarks that—"this double direction in all the superficial and deep parts of the encephalon has not hitherto been the object of an attention proportional to its importance." "There is, however, in the combination of these antero-posterior circles and of rays diverging from the base of the brain to the circumference of all these circles, something so remarkable, that we may, I believe, give it as the character par excellence of the brain of all mammiferæ."—(*L. c.* pp. 350, 356.)

M. Foville then proceeds to explain his views of the signification of the remarkable organization he believes he has thus detected. He contends that the brain contains two elements quite distinct from each other: one of these, which he calls the *cerebral nucleus*, or central cylinder of the brain, is composed of two symmetrical halves anastomosing on the median line, and comprising more especially the corpus callosum, the corpora striata and the optic thalami; the other element consists of the hemispheres, each of which, convex without, is concave within, where it covers the central cylinder or cerebral nucleus, to which it is united by fibrous fasciculi derived from the anterior, inferior plane of the cerebral peduncle or crus. The speculations of the author upon these parts, we prefer giving in his own words. "In considering the ensemble of the parts just described, we cannot avoid feeling the analogy of the central cylinder or nucleus of the brain with the spinal cord. Like the cord, it is hollowed by a ventricular cavity; like the cord, it contains in each of its halves two distinct masses of gray matter, separated the one from the other by layers of white matter; further, this central cerebral cylinder presents, like the cord, an entirely white surface on the exterior. Lastly, we find another point of resemblance between the spinal cord and the central cylinder of the brain, in the two layers of fibrous matter, which unite the hemisphere to the central cylinder; these two fibrous layers represent the two lines of nervous roots, which detach themselves from the surface of the spinal marrow, and converge towards the spinal ganglions. Under this point of view, the central cylinder of the brain is nothing else than the encephalic prolongation of the spinal cord; the cortical layer of the cerebral convolutions would be assimilated with the ganglions developed upon the roots of the spinal nerves; and these roots of the spinal nerves would have, for analogues in the brain, the fibrous layers which in two orders are detached from the central cylinder to the hemisphere."—(*L. c.*, p. 361.) It is difficult to comprehend all the anatomical grounds upon which these speculations rest without referring either to the plates (the most explanatory of which is pl. 13), or, which is of course better, repeating the examination on the brain itself.

Such is a brief summary of the cerebral system, as it may be termed, of Foville, and it is due to this distinguished anatomist to add, that much importance is attached to his researches by some of the most eminent scientific men in France, among whom may be mentioned the names of De Blainville, Dutrochet, and M. Edwards, appointed by the Academy of

Sciences (the Institute), and those of Bouillaud, Bouvier, and Blandin, selected by the Academy of Medicine to report on M. Foville's memoir. But, notwithstanding all these high authorities, we feel assured that almost all the peculiar views of this writer are fundamentally erroneous.

As regards the comparison of the central nucleus with the spinal cord, and that of the hemispheres with the spinal ganglia, we are not exactly aware whether this is intended as a mere morphological or as a physiological analogy; if the latter is indicated, we would observe that, in the present state of knowledge, it must be regarded as a mere surmise, for no experiments have hitherto been performed sufficiently exact to determine either the use of the spinal ganglia or of the corpus callosum, corpora striata, and optic thalami. If the comparison relates only to typical forms, we must remark, that it does not seem to us to rest upon the only safe ground for such hypotheses, namely, developmental and comparative anatomy; and more especially we hold the speculation as to the hemispheres and the spinal ganglia, as totally fallacious. With respect also to the favourite notion of M. Foville, that the corpus callosum is derived from the fibres of the crus cerebri, and not from those of the cerebral hemispheres, we are ourselves satisfied, after an extended observation, that that body is in reality, what it is generally affirmed to be, the great transverse commissure of the brain; and on this point we are happy to be able to quote the words of an eminent anatomist, Mr. Mayo, who, in allusion to the opinion of M. Foville, justly observes, "the cerebrum, which has so many parts in correspondence with parts of the cerebellum, would then want the most striking feature of all, the analogue of the pons Varolii"—a supposition which, when the strictness of the organic laws of formation is recollected, is utterly untenable. There is in addition this great confirmation of the received opinion, the configuration, namely, of the corpus callosum; a vertical section shows it to be thinner in the middle than at the extremities, and thicker at the posterior than at the anterior end—a disposition which is explained by admitting that it receives in the centre the fibres from the hemispheres opposite to that part, whilst at the extremities it collects, in addition, the converging fibres proceeding from the anterior and posterior lobes.

We would further point out to the notice of our readers, that the views both of M. Gerdy and of Foville, respecting the series of rings or annular fibres, are for the most part opposed to the opinions commonly received, especially in this country. The doctrine advocated among us is, that the fornix and the remarkable fasciculus contained within the convolution above the corpus callosum (the part called "l'ourlet" by Foville) are longitudinal commissures, a view supported by our own dissections, and especially by those of Mr. Solly, who has given a bold and accurate representation of these structures. The tænia semi-circularis, and the band described by Foville on the exterior of the corpus striatum, do certainly present the circular disposition; but, as to the raphé of the corpus callosum, its signification is not so clear.

Notwithstanding we have felt it to be a duty thus to express our dissent from so large a portion of M. Foville's conclusions, many of his investigations are of considerable value, especially those which show with so much distinctness the course of the fibres proceeding from the medulla oblongata, and the connexion of the nerves of special sense, the olfactory, the optic, and the auditory, with the prolongation of that portion of the cord which is in relation with the posterior or sentient roots of the spinal nerves : this latter fact, as M. de Blainville points out in his report, gives a powerful support to the theory of Bell. There can, also, be no doubt that a portion of the spinal cord is to be recognised in the interior of the encephalon, including the optic tubercles, the optic thalami, and perhaps the corpora striata, but not, as Foville contends, the corpus callosum: this opinion, however, is not novel.

We have devoted so large a portion of our space to the opinions of M. Foville, that we can only extract from the work of Dr. Todd and Mr. Bowman the following exposition of their views regarding that most complex and important part of the brain—the fibrous apparatus connected with the corpus striatum.

"When thin sections of the corpus striatum are examined by transmitted light, the smallest bundles of fibres observable in them appear to consist of tubules reduced to their minutest dimensions, and closely united to each other. So compactly applied are they, that very little light passes through or between them. Hence they appear to be dark masses lying in the substance of the ganglion, and, from their opacity, it is very difficult to determine their exact relation to the elements of the vesicular matter. Many of the bundles, however, appear to us to attach themselves, at different parts of the ganglion, as if around a large vesicle of which, with its nucleus, we have sometimes seen indications at one extremity of the dark mass of aggregated fibres. Other bundles of fibres appear to emerge from the corpus striatum, and to contribute to form the fibrous matter of the hemisphere."

"Thus, three sets of fibres may be described as existing in the corpus striatum: 1st, those which below enter into the formation of the crus, and above are connected with that ganglion; 2ndly, those which are connected inferiorly with the corpus striatum, and above with the cerebral convolutions; and lastly, those which pass from the white substance of the hemispheres through the corpus striatum to the crus cerebri. And of these three sets of fibres, the first serves to connect the corpora striata with the mesocephale and medulla oblongata; the second to connect the cerebral convolutions with the corpora striata; and the third to connect the convolutions with the mesocephale and medulla oblongata. It must be confessed, however, that the evidence upon which the existence of the third class of fibres rests is less satisfactory than that for the first and second, although most of those anatomists who are contented with coarse dissection seem to recognize only the third class."—*Phys. Anat.* p. 277.

We feel it necessary to express our conviction, resting on repeated and minute dissection, that the third class of fibres, or those proceeding from the crus cerebri, are distinctly continued into the substance of the hemispheres and convolutions.

RELATION OF THE SKULL TO THE BRAIN.—The second division of the volume, lately published by M. Foville, bears upon this question, so much agitated in late years, especially by phrenologists. The author contends that, although the contour of the cranium is modified by the form of the convolutions, this is altogether subordinate to the influence exerted by the lateral ventricles and their prolongations or cornua. The deep investigation of the protuberances of the encephalic box, it is said, does not allow of any doubt upon this point: thus the frontal protuberance corresponding to the anterior horn is, like it, round or oval; the occipital protuberances, but especially the fossæ which correspond to them internally, are like the corresponding part of the ventricle, the posterior cornu, a little more acute than the former; the temporal projections are oblique like the temporal regions of the lateral ventricles; lastly, the convexity of the sincipetal region is perfectly in relation with the arching of the upper surface of the corpus callosum. The influence of the convolutions is, as we have seen, secondary, but still it may considerably modify the external configuration of the skull; if, for example, the convolutions are very large, the cranium maintaining always its general form, which the ventricles impress, swells in the intermediate regions, and consequently the protuberances, are but slightly pronounced; whilst, on the contrary, in persons in whom the convolutions have only a medium development, the frontal, parietal, and occipital protuberances are very apparent. In the first case, to borrow the characteristic expression of Foville, "the form of the cranium is more cerebral than ventricular, whilst, in the second, it is more ventricular than cerebral."—(*L. c.*, p. 102.)

We are inclined to regard these observations as throwing a new light upon the relations existing between the form of the skull and of the surface of the brain; but the evidence of developmental anatomy, which in questions of this kind is of paramount importance, appears to be opposed to the theory of Foville. Most of our readers are doubtless aware that, in the primordial cranium, there exist three distinct divisions or cranial vertebræ, which are unquestionably formed in reference to the three primitive cells into which the portion of the nervous system, contained within the head, is in the embryo divided; but it is found that, although the anterior vertebra or segment, of which the future os frontis is a portion, does correspond with the cerebral hemispheres, and therefore in a general manner with the lateral ventricles, yet the middle vertebra, comprising the future parietal bones, and a part of the sphenoid, corresponds with the corpora quadrigemina; whilst the posterior segment, the future os occipitis, corresponds with the medulla oblongata. Viewing the skull in this way, there appears to be a discrepancy between Foville's investigations and those connected with embryology, which at present cannot be reconciled.

Physiology of the Nervous System.—Having pointed to some of the more interesting and novel facts connected with the anatomy of the nervous system contained in the several works before us, we proceed briefly to notice their contents in relation to physiology. And in the outset it will not be superfluous to state, as a fundamental principle, that all nervous force is restricted to the so-called gray matter, or, to borrow the language of the authors of the Physiological Anatomy, "the vesicular is the true dynamic nervous matter, the source of all nervous power." This position rests upon ample evidence, the principal of which is embodied in the following proofs:—

"1. Nerves, when separated for a time from the nervous centre, lose all power of stimulating their muscles to contraction. No irritation, mechanical or electrical, is sufficient to excite them. If a nerve be divided some distance from the centre, the peripheral portion will, after a time, waste, and lose all power of developing nervous force; but the central portion, which remains in connexion with the centre, retains its nutrition and its vital properties unimpaired.

"2. All nervous centres contain vesicular matter, with which nervous fibres freely intermix.

"3. The power of a nervous centre appears to be proportionate to the quantity of its vesicular matter. This is well exemplified in the cerebral convolutions, the vesicular surface of which is always in the direct proportion of the development of mental power; or, in general terms, *the gray matter increases in the exact ratio of the nervous energy*. (Grainger.)

"4. All nerves appear to arise from vesicular matter. Stilling represents special accumulations of vesicular matter at the origins of the nerves of the medulla oblongata.

"5. Nerves, whose power is exalted for some special purpose, have an increased quantity of gray matter at their origin, of which the electric lobe in the torpedo, connected with the origins of the fifth and eighth pairs of nerves, is an extraordinary instance. (Grainger)."—*Physiol. Anat.* p. 322.

Another truth not less essential is, that the ultimate nervous tubes act as isolated conductors, or, to borrow the words of Valentin, "each peripheral primitive fibre conducts its stimulus, so long as it is confined to the peripheral nerve, entirely in an isolated manner, never transferring it either to a neighbouring or to a remote fibre, whether this be of a similar or heterogeneous nature."—(*L. c.* p. 587.) We regret that our space will not allow us to lay before our readers the clear demonstration of this law, as relates to the sentient and motor nerves, and also to disease, contained in the physiology of this distinguished writer; suffice it to say that, the capability of experiencing local sensation, healthy and morbid, and the power of determining individual muscular actions, involve of necessity the agency of insulated, that is of independent, conductors. Valentin also conceives that, with some very rare exceptions, probably dependent upon their development, the nervous corpuscles act in an isolated manner; at all events these globules are not only separated from one another, but each of them it embedded in its proper sheath. This being the fundamental truth, it is altogether a question of subordinate importance to ascertain how the insulating power is effected. We have ourselves thought it might depend on some property of the neurilemma or investing membrane of the tubule, and this appears to be a more probable explanation than that advanced by Dr. Todd and Mr. Bowman, who conceive that there is "a provision for the insulation of the central axis of each nerve-fibre in the white substance of Schwann," thus making a considerable portion of the neurine itself inactive in innervation. These writers think that the fibres of the sympathetic want the power of insulation possessed by the tubular nerves, an opinion in which, however, we do not concur, and which probably originated in the absence of the white matter of Schwann in these gelatinous fibres.

We are as far as ever from knowing anything of the actual nature of the nervous force. Most physiologists in the present day are satisfied that it is in no way identical with the electric power, a conviction, which, shaken for a time in some quarters by the researches of Dr. W. Philip, was greatly strengthened by the well-contrived experiments of Professor Müller. The deeply interesting discovery of Matteucci might perhaps tend to the revival of the electrical hypothesis, and we therefore think that

Dr. Todd and Mr. Bowman have rendered an acceptable and timely service to the cause of science, by the careful and extended comparison they have instituted between the nervous and electrical forces. After shewing in what points these subtle powers correspond, and in what they disagree, they give these general conclusions :—" The results of experiment certainly afford no support to the advocates of the electrical theory ; and indeed there are difficulties in the way of obtaining the necessary conditions for a satisfactory result, which of themselves invalidate the experiments which have been reported to prove favourable to that theory."

" The proofs, therefore, of the passage of an electric current through the nerve-fibres during nervous action must be held to be altogether defective. Not only is experimental evidence wanting to support the electrical theory, but certain facts are admitted which greatly invalidate it. Of these, a very important one has been adduced in the preceding paragraph. The following may be added, some of which have already been adduced by Müller.

" 1. The firm application of a ligature to a nerve stops the propagation of the *nervous power* below the points of application, but not of *electricity*. The nervous trunk is as good a conductor of electricity after the application of the ligature as before it.

" 2. If a small piece of a nervous trunk be cut out, and be replaced by an electric conductor, electricity will still pass along the nerve, but no nervous force, excited by stimulus above the section, will be propagated through the conductor to the parts below.

" 3. Nervous fibre is not a better conductor of electricity than other tissues. Matteucci assigns to muscle a conducting power four times greater than that of nerve or cerebral matter; and Weber states that no substance in the human body is so good a conductor as the metals. From our own observations on this subject, made with the *most delicate* instruments, we are led to state that both nerve and muscle are *infinitely worse* conductors than copper, and that we have failed in detecting any appreciable difference in the conducting power of these two animal substances."—*L. c.* p. 243.

A similar opinion is expressed by Valentin, who says, in reference to the attempts made at various times to prove the identity of the nervous and the electrical fluids, " all such investigations and observations fall when accurately criticised." He also shows that the phenomena displayed by electrical fishes, when carefully analysed, confirm the conclusion drawn from other sources.

Functions of the Spinal Cord.—This interesting subject is very ably and fully discussed by Dr. Todd and Mr. Bowman, and by Valentin ; Mr. Newport also gives the results of a series of experiments made upon the myriapoda, which confirm the researches made by other physiologists. The two former writers, after adducing various proofs to show that the spinal cord has a power of exciting muscular actions independently of the brain, remark :—

" Some substances exert a peculiar influence upon the spinal cord, and throw it into a state of considerable polar excitement. Strychnine is the most energetic substance of this class. If a certain quantity of this drug be injected into the blood, or taken into the stomach of an animal, a state of general tetanus will quickly ensue, sensibility remaining unimpaired. The slightest touch upon any part of the surface, even a breath of wind blown upon it, will cause a general or partial convulsive movement. The whole extent of the cord is thrown into this polar state, and even the medulla oblongata is involved in it; whence the closed jaws, the spasmodic state of the facial muscles, the difficult deglutition. In this remarkable state of excitement it is curious to observe that the spinal cord is perfectly natural in point of structure, as far as our means of observation enable us to judge. We have examined some spinal cords of animals which have died exhausted by the effects of the strychnine, but have always found the nerve-tubes and other elements of the cord exhibiting their natural appearance." P. 314.

Nothing seems to control this polar state of the cord, which may be induced readily, especially in the frog, so effectually as cold; "ice applied along the spine, or the cold douche, may be frequently employed with great advantage in cases of muscular disturbance dependent upon this polar state of the cord."

One of the most difficult questions in physiology is to determine the offices of the individual columns which we have described as constituting the entire cord; and the addition of at least two new fasciculi—we allude to the narrow lateral bands discovered by Foville—does not render this a more easy task. Our readers need hardly be reminded that the best and latest investigations, especially those of Valentin, Seubert and Longet, have entirely corroborated the great fact announced and proved by Bell, namely, that the anterior root of each spinal nerve is motor, and the posterior sentient. The latter being stimulated will, however, determine muscular contraction upon the excito-motory principle; whilst the former, will produce pain as Magendie had affirmed, and in consequence of an anastomotic filament, which Kronenburg has shown, it derives from the posterior root. But, although the offices of the two roots are determined, it does not follow that the anterior and posterior columns or fasciculi bordering the median fissures, partake in the same properties, and are respectively connected with motion and sensation. If, indeed, "it could be proved that the anterior or motor roots were exclusively connected with the antero-lateral columns, and that the posterior or sensitive ones arose exclusively from the posterior columns, then there would be good anatomical grounds for the doctrine so long erroneously prevalent, that the functions of these columns coincided with those of the roots, that the posterior columns were sensitive and the anterior motor." In opposition to such an inference, we may state that, after a careful examination, we have failed in tracing a single nerve-fibre into either the anterior or posterior fasciculus, all the fibres which do not penetrate into the gray axis, being lost in that lateral part of the cord, which is placed between the anterior and posterior lateral sulci. Neither comparative anatomy nor direct experiment throw much light on this question.

"Nor do we derive much positive knowledge from the researches of the morbid anatomist. Cases, indeed, are on record, which shew that disease of the posterior columns does not necessarily destroy sensibility; that perfect sensibility is compatible with total destruction of the posterior columns in some particular region, the posterior roots remaining intact: and others have occurred in which sensibility has been impaired or destroyed, while the posterior columns remained perfectly healthy. In a remarkable case, related by Dr. Webster, there was complete paralysis of motion in the lower extremities, but sensibility remained; yet there was complete destruction of the posterior columns in the lower part of the cervical region. Similar cases have been put on record by Mr. Stanley and by Dr. W. Budd. Dr. Nasse of Bonn, refers to several cases of the same kind, observed by himself or others. We have ourselves seen two cases in which the prominent symptom was great impairment of the motor power without injury to the sensitive; yet the seat of organic lesion in both was in the posterior columns of the cord. Such a case as that of Dr. Webster's appears to us to be conclusive, so far as the following proposition extends, namely, that sensation may be enjoyed in the inferior extremities *independently of the posterior columns;* and that, even if those columns be sensitive, there must be some other channel for the transmission of sensitive impressions besides them."—*Physiol. Anat.* p. 316.

Dr. Todd and Mr. Bowman suggest that the posterior columns may be in part commissural, uniting the various segments of the cord and so combining their actions, a view which is thought to be supported by the existence in the brain of longitudinal commissures. A stronger argument might, we think, be adduced from the structure of the abdominal (spinal) cord of the articulata, in all of which, though more plainly in some than in others, the individual ganglia are united by longitudinal commissural fibres. These gentlemen thus sum up their conclusions upon this interesting subject.

"The hypothesis, then, which we are most disposed to adopt, is the following:—That *the antero-lateral columns of the spinal cord with the gray matter* are, in connexion with the brain, the recipients of sensitive impressions and volitional impulses, and that they are the centres of the independent or physical nervous actions of the cord; and that *the posterior columns* propagate the influence of that part of the encephalon which combines with the nerves of volition to regulate the locomotive powers, and serve as commissures in harmonizing the actions of the several segments of the cord."—*L. c.* p. 321.

We shall have occasion to refer again to this point in connexion with the functions of the cerebrum.

We shall not enter into the details of the reflex actions, as this subject has been so repeatedly discussed in late years, and especially in the several works of Dr. Marshall Hall. It may, however, be proper to notice that Valentin contends against the existence of a special class of nerves admitted by that distinguished physiologist, under the denomination of *incident* and *reflex.* He says, "such a distinction is not correct, if by it we are to understand the existence of peculiar primitive fibres subservient exclusively to the reflex actions: there are at present no facts sufficient to justify such an hypothesis. So far as we know, every sentient fibre is capable of acting as an incident, and every motor fibre as a reflex fibre." He even goes so far as to deny what may be regarded as a most essential part of Dr. Hall's theory, namely, that the reflex action is the property of the spinal cord, to the exclusion of the cerebral system.—(*L. c.*, p. 763.) We will not, for the reason above stated, allow ourselves to be drawn into this vexed question, and therefore shall only express our entire conviction that, on both the points noticed, Valentin is in error.

Physiology of the Brain.—The portion of Valentin's work treating of the physiology of the brain contains a complete exposition of all the existing knowledge on the subject, and especially of the results obtained by vivisections, but advances little that is new. Although there is no branch of experimental physiology which requires more caution in interpreting results, or which has led to more error and confusion than that which relates to the functions of the several parts of the encephalon, yet many important facts have by this means been obtained, and among the rest, the remarkable one that the hemispheres both of the cerebrum and cerebellum, the seat of all sensation, are themselves insensible to contact. On this subject Valentin thus expresses himself: "Considered theoretically, we should expect that the sentient fibres, which proceed from the medulla oblongata, and expand themselves in all parts of the greater and lesser brain, would bestow on these formations, as well as upon medulla oblongata, a high degree of sensibility. But experience gives results for which a satisfactory explanation is still entirely deficient: thus, if the cerebral hemispheres be laid bare in a mammal or bird, an operation which in itself in no degree destroys the capability of perceiving pain, we find that they can be touched and even transfixed without in the least disturbing the animal; it only struggles and cries out when the trifacial nerve, the crura cerebri, the optic thalami, or the medulla oblongata, are accidentally touched. Again, if the hemispheres be removed by slices down to the centrum ovale, or to the cavity of the lateral ventricle, the animal remains as indifferent as if we were cutting a hair or a nail. The same phenomena have also been repeatedly observed in man; thus, a portion of the hemisphere projecting through a wound of the skull has been removed without producing any action; and, again, parts of the substance of the hemisphere have been taken away by the surgeon in removing pus or foreign bodies without the patient's consciousness."—(*L. c.*, p. 743.)

We have given this extract prefatory to considering that most difficult, and yet most fundamental, question—*what is the true organ or seat of sensation and volition?* The phenomena just stated, and more especially the well-known experiments of Magendie, have induced many physiologists to fix upon the medulla oblongata and pons Varolii; but, among many other insuperable objections to this opinion, we may urge with Valentin that, "were it correct, neither the crus cerebelli nor the crus cerebri ought to cause, when irritated, painful sensations." The authors of the Physiological Anatomy, undeterred by the failures of Magendie, Saucerotte, Foville, Longet, and a multitude of other writers, have ventured, with more detail and precision, to define the local habitations of volition and sensation. They observe, in speaking of the medulla oblongata, pons Varolii, corpora striata, and optic thalami, that, "although the anatomist may find it convenient to describe these parts each by itself, it is impossible, in the consideration of their functions, to separate them completely, they are so closely connected with each other, and the functions of one part are so readily affected by any change in those of the other. Thus, the olivary columns, which form the central and most essential part of the medulla oblongata, extend upwards through the mesocephale to the optic thalami; and the anterior pyramids form an intimate connexion not only with the vesicular matter of the mesocephale, but, to a great extent, with that of the corpora striata. All these parts taken together, with the quadrigeminal tubercles, will be found to be the centre of the principal mental nervous actions, and of certain physical actions which are very essential to the integrity of the economy."—(*L. c.*, p. 342.)

Then follows an investigation of the individual offices of these parts. After alluding to the medulla oblongata as the channel through which the brain operates on the cord to produce voluntary motion, and as the "primum movens" of respiration and deglutition, the authors remark, "it seems not improbable that the centre of volition is connected with one or both of the gangliform bodies (corpora striata and optic thalami) in which the columns of the medulla oblongata terminate above. When the cerebral hemispheres have been removed, as in Flourens' and in Magendie's experiments, the bird is thrown into a deep sleep, a state of stupefaction, and insensibility to surrounding objects. But he can maintain his attitude—stand—walk, when first propelled—fly, if thrown into the air. This continuance of the locomotive power implies some degree at least of mental or volitional effort. All the animal's movements have much of the appearance of the exercise of will, although, doubtless, many of them are in a great degree excited by physical stimuli. Hence there seems reason to believe that the will exerts a primary influence upon either or both of these gangliform bodies, more vigorous when aided and guided by the power of the cerebral hemispheres. The frequent paralysis of motion apart from sensation, when the upward continuations of the pyramidal fibres in the corpora striata are diseased, renders it extremely probable that these fibres are the media of connexion between the brain and cord in voluntary actions;" and it is subsequently added, "the same line of argument which leads us to view the corpora striata as the more essential parts of the nervous apparatus which control direct voluntary movements, suggests that the optic thalami may be viewed as the principal foci of sensibility, without which the mind could not perceive the physical change resulting from a sensitive impression. The principal anatomical fact which favours this conclusion, is the connexion of all the nerves of pure sense, more or less directly with the optic thalami, or with the olivary bodies."—(*L. c.*, p. 349.)

In connexion with this subject, Dr. Todd and Mr. Bowman have some peculiar views respecting the share which the cord takes in volition and sensation, and on the mode in which its action is called into play; their theory, indeed, is so novel, that no apology is necessary for laying before our readers the following extracts, in which it is embodied.

"The mechanism of a voluntary action, in parts supplied with spinal nerves, would be, according to this hypothesis, as follows: The impulse of volition, primarily excited in the brain, acts at the same time upon the gray matter of the cord (its anterior horn), which in virtue of its association with the former, by means of the fibres of the anterior pyramids, becomes part and parcel of the organ of the will, and therefore as distinctly amenable to acts of the mind as that portion which is contained within the cranium." P. 330.

"An objection to this explanation will readily be raised, that the excitation of the anterior horn of the gray matter, in the way stated, does not explain the remarkable power which the will has of *limiting* its action to one or two, or a particular class of muscles. We reply to this, however, that there can be no reason for denying to the mind the faculty of concentrating its action upon a particular series of the elementary parts of the vesicular matter, or even upon one or more vesicles, if we admit that it can direct its influence to one or more individual fibres, as the advocates of the first and second hypotheses do. If, indeed, we admit the one, we must admit the other; for whether the primary excitation of a fibre take place in the encephalon or in the spinal cord, the part first affected must probably be (according to our second postulate) one or more vesicles of the gray substance.

"The series of changes which would develope a sensation, admits of the following explanation: A stimulus applied to some part of the trunk or extremities is propagated by the sensitive nerves to the posterior horn of the gray matter of the spinal cord, and from the junction of this part with the brain either through the direct continuity of the vesicular matter of the cord with that of the centre of sensations, or through longitudinal commissural fibres, analogous to, or even perhaps forming part of, the anterior pyramids, this organ is simultaneously affected." P. 330.

"It is not necessary to the development of sensation that the fibre stimulated should be implanted directly in the brain; if it be connected with this centre through the medium of vesicular matter of the same character as that which is found in it or through commissural fibres, all conditions necessary for the development and propagation of nervous force would appear to be fulfilled. It must not be supposed, however, that in making this statement we mean to assign the spinal cord to be the *seat* of sensation; all we assert is, that the posterior horns of its grey matter, as being the part in which the sensitive roots are implanted, participate largely in the mechanism of sensation; and that by their union with the brain they become, *pro tanto*, a part of the centre of sensation, so long as that union is unimpaired.

"This hypothesis offers an explanation of the hitherto unexplained phenomenon of impaired sensation on that side of the body which is opposite to the seat of cerebral lesion. If we regard the anterior pyramids as commissures between the sensitive, as well as between the motor portions of the cerebro-spinal centre, it will be obvious that the posterior horns of the spinal gray matter on the right side will be associated with the left centre of sensation in the brain, and *vice versâ*.—*L. c.*, p. 331.

The writers afterwards remark, with respect to the hemispheres, "the convolutions of the brain are *the centre of intellectual action*, or, more strictly, this centre consists in that vast sheet of vesicular matter which crowns the convoluted surface of the hemispheres. This centre is connected with the centres of volition and sensation (corpora striata and optic thalami), and is capable at once of being excited by, or of exciting them." —*L. c., p.* 365.

Having thus afforded our readers the means for forming their own opinion, we must say for ourselves, that, although highly esteeming the acquirements of the authors, we can in no degree coincide with the hypotheses above stated. So far indeed from agreeing with these gentlemen, we feel it necessary to express our decided conviction, that their views, being opposed both to the principles which govern the nervous system, and to the evidence of comparative anatomy, would, if adopted, tend to obstruct the progress of physiology in one of its most interesting branches. Our limits will not permit us to go into all the evidence on which we rest our opinion, and therefore we must confine ourselves to a very few general remarks. And, in the first place, we would observe, that it is objectionable in principle to admit of three distinct and independent centres of consciousness, (intelligence, volition and sensation being only forms of consciousness); for, if such were really the case, three independent sentient existences would be bound up in every animal, a condition which seems opposed to the idea of personal identity and individuality. The circumstance on which so much reliance is placed, of a pure nerve of sense, the optic, being attached to the thalamus, is no more an argument of that gray mass being the centre of sensation, than is the attachment of the posterior sentient root of a spinal nerve a proof that the corresponding segment of the spinal marrow is a centre of feeling: in both these instances, as far as consciousness is concerned, the thalamus and the cord act only as conductors transporting the impression towards the hemispheres, where alone, as we contend, in all cases the act of perception is consummated. This conclusion is further supported by the fact, that the cord has its own peculiar powers, seen in full operation in anencephalous infants who have survived their birth; and also by the circumstance that, when in injury of the cervical region of the spine, the larger portion of this so-called instrument of sensation is rendered inoperative, the power of consciousness and feeling, instead of being weakened or impaired, remains intact.

We will only notice one more point, and it is this:—the hypothesis of the thalamus being the seat of sensation, receives, as it is affirmed, much support from the anatomy of the brain in the articulata, among which the optic nerve is, in the perfect animal, attached to the supra-œsophageal ganglion, which body, the unquestionable and sole organ of consciousness in these creatures, is thus said to represent the optic thalamus of the vertebrata. It is certain, however, that the brain in the invertebrata is more complex than it is usually conceived to be; a position supported by the valuable dissections of Mr. Newport (*see Med. Chir. Rev, July* 1845, *p.* 11), who has found, for example, that in the myriapoda the brain is, in the embryo, composed of four pairs of ganglia, of which the second pair receive the optic nerves, whilst those of the antennæ are attached to the first pair. Now, we hold the first pair of ganglions, with the antennal nerves attached, like the olfactory in the vertebrata, as constituting the true organ of consciousness; and thus the encephalic organization of the articulata entirely corresponds with the theory that the cerebral hemispheres are in all classes the true organ of consciousness.

There being so much difficulty in assigning the office of the individual parts of the encephalon, it would be an unprofitable occupation to inquire further whether the corpora striata have a special influence upon the motions of the lower extremities, and the thalami upon those of the upper; all these questions, when experimentally prosecuted, involve such destructive mutilations of the brain and other mischief, that they are never likely to be cleared up by vivisections; we must rather look to the more satisfactory evidence derived from pathology and comparative anatomy.

Our notice has been already so much extended, that we must dismiss the papers of Professor Bidder and Dr. Bernard with a few observations. The former physiologist, some few years ago, published, in conjunction with Professor Volkmann, an interesting work to prove, on anatomical grounds, that the sympathetic nervous system was independent of the brain and spinal cord. In a paper contained in Müller's Archives of last year, he relates some experiments made on frogs, in order to support physiologically the same conclusion. He found that the animals survived the loss of the brain alone ten or fourteen days; if the cord was also destroyed, leaving, however, the medulla oblongata so as to preserve respiration, the frogs lived five or six days; and when the whole of the brain and cord were removed, the animals lived till the second day. The circulation of the blood in the swimming membrane continued after all these mutilations, and as this proved the persistence of the power of the heart, it must also be received as evidence that the great sympathetic has an energy of its own, unless indeed, which few will maintain, it be assumed, that the circulation is alogether independent of the nervous system. The secretion of urine likewise continued after the destruction of the brain and spinal cord, indicated by an accumulation in the bladder.—(*L. c.*, p. 376.) Some experiments are also related to show that the secretion of the gastric juice and the solution of food persisted, but the evidence of this is unsatisfactory. So far as these experiments upon the frog may be relied on, the inference is, that the ganglionic portion of the nervous system constitutes an independent power, a conclusion which is further supported by reasoning deduced from anatomy.

Dr. Bernard's researches relate to a point on which there exists much contrariety of opinion among physiologists—the true function of the spinal accessory nerve of Willis, and its relation to the pneumo-gastric : is the accessory a nerve of motion, or of sensation, or of both—is it a nerve of volition or of the involuntary class? Some years ago, Dr. Bischoff, of Heidelberg, performed some experiments which convinced him that the accessory was the motor or anterior root of the vagus nerve ; but he has since abandoned this opinion, and adopted the views prevailing among German physiologists, that it is in office a compound nerve of motion and sensation. Bernard points out the necessity of distinguishing between respiration and the voice, which, although they seem to be anatomically confounded, are physiologically independent, and are exercised, as he contends, under antagonistic and distinct nervous influences, although the muscles are in common.—(*Archiv. Gener. de Medicine*, t. 5, p. 68.) He further believes he has shown by experiments, that whilst the pneumogastric is in itself a mixed nerve which governs the motor and sentient phenomena of the three great organic functions, respiration, digestion, and circulation, the accessory is purely a motor nerve, which in a remarkable manner governs the movements of the larynx and of the thorax at all times when those organs are engaged in actions beyond simple respiration, as in the production of the voice, of efforts, &c. In these actions the larynx is vocalized by the internal branch of the accessory, and the thorax by the external branch, so that if the former be divided, the animal, when it attempts to cry, produces no sound, but only executes respiratory movements more active than usual ; whilst, if the latter be cut, the larynx retains its power of producing the vocal sound, but the thorax loses its power of extending or modulating it. M. Bernard thus confirms the important distinction first established by Bell between those actions of the cervical and thoracic muscles which are connected with respiration proper and those which are productive of voice ; but he has thrown a new light on the nerves interested, for, whilst our illustrious countryman regarded the accessory as of a respiratory, and hence involuntary character, Bernard conceives that it is strictly a nerve of volition. These researches appear to have been conducted with great care, and as they bear on a question of prime import in neurology—the endowments of the respiratory nerves, they are well worthy the attention of physiologists.

